# Excitotoxic neuronal cell death during an oligodendrocyte-directed CD8^+^ T cell attack in the CNS gray matter

**DOI:** 10.1186/1742-2094-10-121

**Published:** 2013-10-05

**Authors:** Nico Melzer, Gordon Hicking, Stefan Bittner, Nicole Bobak, Kerstin Göbel, Alexander M Herrmann, Heinz Wiendl, Sven G Meuth

**Affiliations:** 1 Department of Neurology, University of Münster, Albert-Schweitzer-Campus 1, Münster 48149, Germany; 2 Department of Physiology I - Neuropathophysiology, University of Münster, Robert-Koch-Straße 27a, Münster 48149, Germany

**Keywords:** Glutamate excitotoxicity, Neuronal cell death, CNS tissue damage, CD8^+^ T cells

## Abstract

**Background:**

Neural-antigen reactive cytotoxic CD8^+^ T cells contribute to neuronal dysfunction and degeneration in a variety of inflammatory CNS disorders. Facing excess numbers of target cells, CNS-invading CD8^+^ T cells cause neuronal cell death either via confined release of cytotoxic effector molecules towards neurons, or via spillover of cytotoxic effector molecules from 'leaky’ immunological synapses and non-confined release by CD8^+^ T cells themselves during serial and simultaneous killing of oligodendrocytes or astrocytes.

**Methods:**

Wild-type and T cell receptor transgenic CD8^+^ T cells were stimulated *in vitro*, their activation status was assessed by flow cytometry, and supernatant glutamate levels were determined using an enzymatic assay. Expression regulation of molecules involved in vesicular glutamate release was examined by quantitative real-time PCR, and mechanisms of non-vesicular glutamate release were studied by pharmacological blocking experiments. The impact of CD8^+^ T cell-mediated glutamate liberation on neuronal viability was studied in acute brain slice preparations.

**Results:**

Following T cell receptor stimulation, CD8^+^ T cells acquire the molecular repertoire for vesicular glutamate release: (i) they upregulate expression of glutaminase required to generate glutamate via deamination of glutamine and (ii) they upregulate expression of vesicular proton-ATPase and vesicular glutamate transporters required for filling of vesicles with glutamate. Subsequently, CD8^+^ T cells release glutamate in a strictly stimulus-dependent manner. Upon repetitive T cell receptor stimulation, CD25^high^ CD8^+^ T effector cells exhibit higher estimated single cell glutamate release rates than CD25^low^ CD8^+^ T memory cells. Moreover, glutamate liberation by oligodendrocyte-reactive CD25^high^ CD8^+^ T effector cells is capable of eliciting collateral excitotoxic cell death of neurons (despite glutamate re-uptake by glia cells and neurons) in intact CNS gray matter.

**Conclusion:**

Glutamate release may represent a crucial effector pathway of neural-antigen reactive CD8^+^ T cells, contributing to excitotoxicity in CNS inflammation.

## Introduction

Cytotoxic CD8^+^ T cells directed against neuronal or glial antigens contribute to neuronal cell death in a variety of inflammatory CNS disorders [1-3]. They are capable of harming neuronal function and integrity either by directly targeting neurons and their neurites or by exerting their action via 'collateral’ mechanisms of neuronal damage that might follow destruction of oligodendrocytes (ODCs), myelin sheaths or astrocytes in the CNS gray and white matter [4].

Recently, we have shown that activated T cell receptor (TCR)-transgenic, ovalbumin (OVA)-reactive CD8^+^ T cells (OT-I cells [5]) are capable of initiating 'collateral’ neuronal apoptosis in the gray matter of otherwise non-inflamed acute brain slices from ODC-OVA mice selectively expressing OVA in ODCs under the control of a truncated myelin basic protein (MBP)-promotor [6]. 'Collateral’ killing of neurons by OT-I cells was in part due to release of perforin (and granzymes) whereas Fas-ligand (FasL) - Fas-receptor (Fas) interactions were not relevant [7-9]. However, a certain part of the collateral neuronal cell death induced by CD8^+^ T cells remained unexplained [7,10].

During culture of polyclonally activated CD3^+^ T cells, high levels of extracellular glutamate have been shown to accumulate, which are efficiently cleared if T cells are co-cultured with astrocytes [11]. In turn, the T cell-derived glutamate elicits the release of thiols (cysteine, glutathione and cysteinyl-glycine) and lactate from astrocytes, which reduce neuronal apoptosis induced by oxidative stress [11]. However, whether glutamate derived from T cells migrating through the intact CNS parenchyma and scanning for their cognate antigen may also exert excitotoxic neuronal cell death, is unclear at present.

## Methods

### Mice

Wild-type (WT), ODC-OVA [6] and OT-I [5] mice were kept at the animal facility of the University of Münster, Germany, and had access to food and water *ad libitum*. All experiments were conducted according to the German law of animal protection and were approved by local authorities. All transgenic mice were created on or back-crossed to a C57BL/6 genetic background for at least ten generations.

### Isolation of splenocytes and stimulation of CD8^+^ T cells

Spleens of WT or OT-I mice were removed and single cell suspensions were generated by mashing spleens through a 70 μm strainer and lysing red blood cells with ammonium-chloride-potassium (ACK) buffer. Splenocytes were cultured in glutamine-containing DMEM-based standard medium consisting of DMEM (BioWhittaker, Verviers, Belgium) supplemented with 5% FCS (PAA Laboratories, Pasching, Germany), 10 mM 4-(2-hydroxyethyl)-1-piperazineethanesulfonic acid (HEPES, Gibco, Invitrogen, Darmstadt, Germany), 2 mM L-glutamine (PAA Laboratories, Pasching, Germany), 50 μM 2-mercaptoethanol (Gibco, Invitrogen, Darmstadt, Germany), 1% non-essential amino acids (BioWhittaker, Verviers, Belgium) and 25 μg/ml gentamicin (Gibco, Invitrogen, Darmstadt, Germany).

For *in vitro* T cell stimulation experiments, CD8^+^ T cells were isolated from WT splenocytes using the respective MACS^®^ T cell isolation kit (Miltenyi, Bergisch Gladbach, Germany) according to the manufacturer’s instructions. This yielded a purity of about 90% of CD3^+^ CD8^+^ T cells (Additional file 1: Figure S1A, B). CD8^+^ T cells from WT mice were cultured for different periods of time at a density of 1 × 10^6^/well on 24-well plates with anti-CD3/CD28 beads (Dynal Biotech, Oslo, Norway) at different bead-to-cell ratios in the absence or presence of certain glutamate release blockers (all from Sigma-Aldrich, München, Germany) or were left unstimulated. In a subset of experiments, CD8^+^ T cells from WT mice were stimulated in glutamine-free DMEM-based medium (BioWhittaker, Verviers, Belgium). After three days of stimulation, supernatants were removed and analyzed for IFNγ using a mouse-IFNγ-ELISA kit according to the manufacturer’s protocol (Duoset, R&D Systems, Wiesbaden, Germany).

For *in vitro* T cell stimulation experiments, splenocytes from OT-I mice were plated at a density of 1 × 10^7^/well on 12-well plates and primed by incubation for five days with OVA-peptide_257-264_ (SIINFEKL; 1 nM) and IL-2 (500 U/ml). On day three, supernatants were removed, analyzed for IFNγ using a mouse-IFNγ-ELISA kit according to the manufacturer’s protocol (Duoset, R&D Systems, Wiesbaden, Germany) and substituted by fresh medium. On day four, again 500 U/ml of IL-2 were added to the medium. In a subset of experiments, OT-I T cells were repetitively stimulated by incubation with OVA-peptide_257-264_ (SIINFEKL; 1 nM) and IL-2 (500 U/ml) every three days for a total of fifteen days.

### Flow cytometry

Before and after various time periods of *in vitro* culture, flow cytometry of stimulated WT CD8^+^ T cells and OT-I cells was performed using standard methods. For analysis of T cell subtype distribution, cells were stained for 20 minutes with allophycocyanin (APC)-labeled anti-mouse CD3, phycoerythrin (PE)-labeled anti-mouse CD8 and fluorescein isothiocyanate (FITC)-labeled anti-mouse CD4 (all BD Bioscience, Heidelberg, Germany). As isotype controls, cells were stained with APC-labeled anti-mouse IgG1, PE-labeled anti-mouse IgG1 or FITC-labeled anti-mouse IgG1 (all by BD Bioscience, Heidelberg, Germany). For analysis of T cell activation markers, cells were stained for 20 minutes with FITC-labeled anti-mouse CD25 or FITC-labeled anti-mouse CD69 (all BD Bioscience, Heidelberg, Germany). For analysis of T cell viability, cells were stained for 20 minutes with FITC-labeled anti-mouse Annexin V (BD Bioscience, Heidelberg, Germany) and propidium iodide (PI). All antibodies were titrated for optimal staining. Flow cytometry analysis was performed using a FACSCalibur^®^ system (BD Biosciences, Heidelberg, Germany) and results were analyzed using CellQuest Pro Software (BD Bioscience, Heidelberg, Germany).

### Determination of supernatant glutamate levels

Supernatant glutamate levels were determined at different time points of *in vitro* stimulation of WT CD8^+^ T cells and OT-I cells using a commercially available enzymatic determination kit (Glutamine-Glutamate Kit, Sigma-Aldrich, München, Germany) following manufacturer’s instructions. Background glutamate levels were determined in the absence of cells and subtracted from those obtained during incubation and stimulation of cells. All pharmacological blockers used did not interfere with measurements of fixed concentrations of glutamate.

For estimation of single cell glutamate secretion rates, splenocytes from OT-I mice were plated at a density of 1 × 10^7^/well on 12-well plates and iteratively stimulated by incubation for time intervals of 72 hours with OVA-peptide_257-264_ (SIINFEKL; 1 nM) and IL-2 (500 IU/ml) for a total of 15 days. Numbers (N) of CD8^+^ T cells were determined by resuspending and counting total cell numbers/well at the beginning (b) and the end (e) of each interval and multiplying each by the percentage of CD3^+^ CD8^+^ T cells as determined by standard flow cytometry. Cumulative glutamate concentrations ([Glu]) were determined at the end (e) of each time interval as described. Volumes (V) of the culture medium were determined at the beginning (b) and the end (e) of the individual time intervals. Single cell glutamate secretion rates (∆Glu/∆t/CD8 T cell) during repetitive stimulation were calculated using the following formula:

$$\frac{\frac{\Delta \text{Glu}}{\Delta t}}{\mathrm{CD}8\text{Tcell}}=\frac{\left[\text{Glu}\right]e\times \left(\frac{\mathrm{Vb}+\mathrm{Ve}}{2}\right)}{\frac{\left(N\mathrm{CD}8\mathrm{Tcells}\right)b+\left(N\mathrm{CD}8\mathrm{Tcells}\right)e}{2}}$$

### Quantitative real-time PCR

For analysis of glutaminase-, vesicular (V-type) proton ATPase-, and vesicular glutamate transporter (VGluT)1-3-mRNA expression regulation, RNA was purified using Trizol reagent (Invitrogen, Carlsbad, CA, USA) and complementary DNA (cDNA) synthesis was performed using a standard protocol with random hexamer primers (all reagents were purchased from Applied Biosystems, Foster City, CA, USA) from WT CD8^+^ T cells before and after three days of anti-CD3/CD28 bead-stimulation. cDNA was used in quantitative real-time PCR (qRT-PCR) assays with specific TaqMan^®^ primers and probes for murine glutaminase (Mm01257297_m1), V-type proton ATPase (Mm01222963_m1), VGluT1 (Mm00812886_m1), VGluT2 (Mm00499876_m1) and VGluT3 (Mm00805413_m1, all Applied Biosystems, Foster City, CA, USA) as well as primers for 18sRNA (Hs_4319413E; Applied Biosystems, Foster City, CA, USA) as endogenous control. qRT-PCR was performed according to the manufacturer’s protocol.

### Neuronal cell culture and glutamate excitotoxicity assays

Neuronal cell cultures were obtained from WT C57BL/6 mice embryos (E18) following previously described protocols [12]. Pregnant mice were killed by cervical dislocation and embryos were removed and transferred into warmed Hank’s balanced salt solution (HBSS, Gibco, Darmstadt, Germany). After preparation of hippocampi, tissue was collected in 5 ml of 0.25% trypsin in HBSS. After five minutes of incubation at 37°C, tissue was washed twice with HBSS and dissociated in 1 ml of neuronal medium (10% 10× modified Earl’s medium (MEM), 0.2205% sodium bicarbonate, 1 mM sodium pyruvate, 2 mM L-glutamine, 2% B27 supplement (all Gibco, Darmstadt, Germany), 3.8 mM glucose (Merck, Darmstadt, Germany), 1% penicillin/streptomycin (Biochrom AG, Berlin, Germany)) by triturating with fire polished Pasteur pipettes of decreasing tip diameter. Neurons were diluted in neuronal medium and plated at a density of 60,000 cells/cm^2^ on poly-D-lysine (Sigma-Aldrich, München, Germany) coated cover-slips placed in four-well plates (Nunc, Roskilde, Denmark). All neuronal cell cultures were maintained at 37°C and 5% CO_2_ for up to five to seven days prior to experiments. The neuronal cell culture system exhibited a purity of about 80% [12].

After experiments, neurons were fixed with 4% paraformaldehyde (PFA, Merck, Darmstadt, Germany), washed three times with 10 mM PBS, and incubated for seven hours at 4°C in 10 mM PBS containing 5% BSA, (Sigma-Aldrich, München, Germany), 0.2% Triton X-100 (Sigma-Aldrich, München, Germany) and 1% normal goat serum. Slices were then incubated with antibodies to NeuN (1:1,000, Chemicon, Billerica, MA, USA) or MAP2 a/b (1:200, Abcam, Cambridge, UK) and activated caspase-3 (1:400, Cell Signaling, Boston, MA, USA) overnight at 4°C. Secondary antibodies were Alexa Fluor 488-coupled goat anti-mouse IgG (Invitrogen, Carlsbad, CA, USA) and Cy3-coupled goat anti-rabbit IgG (Dianova, Hamburg, Germany) (one hour, RT). Negative controls were obtained by either omitting the primary or secondary antibody and gave no signal (data not shown). Finally, cultures were washed three times and subsequently covered with ProLong Gold Antifade Reagent with DAPI (Invitrogen, Carlsbad, CA, USA). Pictures were collected by immunofluorescence microscopy (Axiophot; Zeiss, Oberkochen, Germany).

Analysis of excitotoxic neuronal cell damage was performed via two distinct experimental approaches: (i) cultured neurons were incubated for 30 minutes with standard artificial cerebrospinal fluid (ACSF, for composition see below) supplemented with different concentrations of L-glutamate (Sigma-Aldrich, München, Germany) followed by a washout for 30 minutes with standard ACSF. The proportion of neurons showing focal swellings (beading [13]) was calculated as a percentage of all MAP2^+^ cells; (ii) cultured neurons were incubated for six hours with standard ACSF (for composition see below) supplemented with or without L-glutamate (Sigma-Aldrich, München, Germany) at a concentration of 100 μM. The proportion of neurons staining positive for activated caspase-3 was calculated as a percentage of all NeuN^+^ cells.

### Cytotoxicity assay

50,000 EL-4 cells/well [14] were cultured on white 96-well microassay plates (Greiner Bio-One, Frickenhausen, Germany) and incubated with or without OVA-peptide_257-264_ (SIINFEKL; 1 nM) for six hours. Alternatively, 50,000 EG-7 cells/well [15] constitutively expressing OVA were cultured on white 96-well microassay plates (Greiner Bio-One, Frickenhausen, Germany). Afterwards, EL-4 or EG-7 cells were co-cultured with 50,000 activated OT-I T cells/well (1:1) in the absence and presence of the N-methyl-D-aspartate (NMDA-) receptor antagonist (+)-5-methyl-10,11-dihydro-5H-dibenzo[a,d]cyclohepten-5,10-imine maleate (MK-801; 10 μM, Sigma-Aldrich, München, Germany) and the L-alpha-amino-3-hydroxy-5-methyl-4-isoxazole propionate (AMPA-)/kainate-receptor antagonist 2,3-dihydroxy-6-nitro-7-sulphamoyl-benzo(F)quinoxaline (NBQX; 30 μM, Sigma-Aldrich, München, Germany) for six hours. The amount of ATP in the supernatant following cell lysis was assessed as a parameter of cell viability and number using the ATPLite™ Luminescence Assay System (PerkinElmer, Rodgau-Jügesheim, Germany) according to the manufacturer’s instructions. Luminescence was measured on a Topcount NXT (PerkinElmer, Rodgau-Jügesheim, Germany). Experiments were performed in triplicates. As a control, supernatant ATP levels were determined from 0, 50,000 and 100,000 EL-4 cells or activated OT-I T cells/well cultured separately for six hours. Under all conditions, supernatant ATP levels showed a linear dependence on the number of cultured cells.

**Figure 1:**
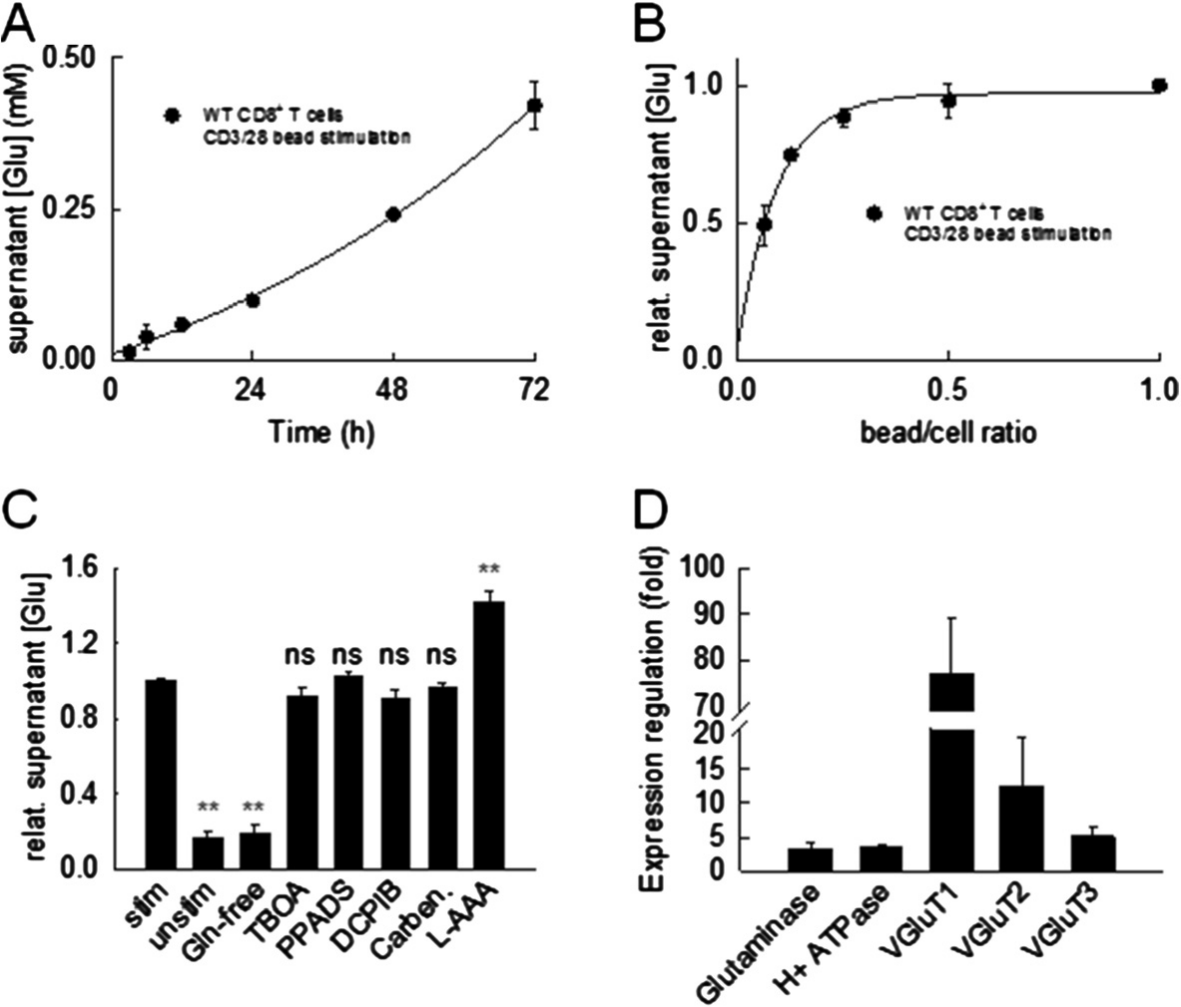
**Activated CD8**^**+ **^**T cells release glutamate putatively by a vesicular mechanism. (A**, **B)** Supernatant glutamate concentrations rise exponentially with time (A; bead-to-cell ratio of 1:1, medium containing 2 mM glutamine; n = 3 mice, experiments performed in triplicates) and depend on the bead-to-cell ratio as a measure of stimulation intensity (B; stimulation for 72 hours, medium containing 2 mM glutamine; n = 3 mice, experiments performed in triplicates) during polyclonal stimulation of WT CD8^+^ T cells with anti-CD3/28 beads *in vitro*. **(C)** Glutamate liberation by WT CD8^+^ T cells depends on extracellular glutamine (Gln) and is not mediated by a known non-vesicular release mechanism (that is, system X_c_-glutamate-cystine exchanger blocked by L-AAA (2.5 mM), volume-sensitive anion channels blocked by DCPIB (5 μM), excitatory amino acid transporters blocked by TBOA (500 μM), connexin hemi-channels blocked by carbenoxolone (10 μM), or P_2_× receptors blocked by PPADS (100 μM); stimulation for 72 hours at a bead-to-cell ratio of 1:1, medium containing either 2 or 0 mM glutamine; n = 3 mice, experiments performed in triplicates). **(D)** Consistent with a vesicular mechanism of glutamate liberation, activated WT CD8^+^ T cells upregulate expression levels of glutaminase, vesicular proton ATPase (H^+^ ATPase), and vesicular glutamate transporters (VGluT 1 to 3) as revealed by quantitative RT-PCR (n = 3 mice, experiments performed in triplicates).

### Preparation of acute brain slices and co-culture experiments with CD8^+^ T cells

Six- to eight-week old ODC-OVA mice [6] were anesthetized with isoflurane and decapitated, the brain was removed and placed in ice-cold saline containing: sucrose, 200 mM; piperazine-N,N’-bis(2-ethanesulfonic acid) (PIPES), 20 mM; KCl, 2.5 mM; NaH_2_PO_4_, 1.25 mM; MgSO_4_, 10 mM; CaCl_2_, 0.5 mM; dextrose, 10 mM; pH 7.35, adjusted with NaOH. Slices were prepared as 300 μm coronal sections on a vibratome [7]. For incubation with CD8^+^ T cells, slices were transferred each into a well of a 12-well plate containing standard ACSF containing (mM): NaCl, 125 mM; KCl, 2.5 mM; NaH_2_PO_4_, 1.25 mM; NaHCO_3_, 24 mM; MgSO_4_, 2 mM; CaCl_2_, 2 mM; dextrose, 10 mM; pH adjusted to 7.35 by bubbling with a mixture of 95% O_2_ and 5% CO_2_. Each slice was incubated with 5 × 10^5^ activated OT-I T cells for six hours in the absence and presence of MK801 (10 μM) and NBQX (30 μM). Afterwards slices were harvested and embedded using OCT compound tissue-tek (Sakura Finetek Europe, Zoeterwoude, Netherlands) and frozen in liquid nitrogen. Ten micrometer coronal cryo-sections were obtained using a cryostat (Leica CM 1950, Wetzlar, Germany).

### Immunofluorescence-staining and -microscopy

Immunohistochemical staining was performed on 10 μm coronary sections. For double labeling, slices were postfixated in 4% PFA for ten minutes and afterwards incubated in blocking solution (PBS containing 5% BSA, 1% normal goat serum and 0.2% Triton X-100). Slices were then incubated simultaneously with antibodies to NeuN (1:1,000, Chemicon, Billerica, MA, USA) and activated caspase-3 (1:400, Cell Signaling, Boston, MA, USA) overnight at 4°C. Secondary antibodies were Alexa Fluor 488-coupled goat anti-mouse IgG and Cy3-coupled goat anti-rabbit IgG. Negative controls were obtained by either omitting the primary or secondary antibody and gave no signal (data not shown). For quantification of cell densities, sections were examined with an Axiophot2 microscope (Zeiss, Oberkochen, Germany) with a CCD camera (Visitron Systems, Tuchheim, Germany). Cell densities were determined in preselected fields within the neocortex.

### Reagent preparations

L-aminoadipic acid (L-AAA), 4-(2-butyl-6,7-dichloro-2-cyclopentylindan-1-on-5-yl)oxybutyric acid (DCPIB), L-β-threo-benzyl-aspartate (TBOA), carbenoxolone (carben.), pyridoxal phosphate-6-azo(benzene-2,4-disulfonic acid) (PPADS), (+)-5-methyl-10,11-dihydro-5H-dibenzo[a,d]cyclohepten-5,10-imine maleate (MK801) and 2,3-dihydroxy-6-nitro-7-sulphamoyl-benzo(F)quinoxaline (NBQX; all Sigma-Aldrich, München, Germany) were dissolved in H_2_O or DMSO as applicable and frozen as aliquots for further use. The solvent solution in the final experimental solution did not exceed 0.1%.

### Statistical analysis

All results are presented as mean ± standard error of the mean (SEM). Statistical analysis was performed using student’s *t*-test for normally distributed samples or Mann–Whitney test for not-normally distributed datasets [16]. A Bonferroni-corrected ANOVA was used in case of multiple comparisons. Analysis was performed using the Sigma Plot 11.0^®^ software (Systat Software Inc., Erkrath, Germany). *P*-values < 0.05 were considered statistically significant (indicated as *) and *P*-values < 0.01 were considered highly statistically significant (indicated as **).

## Results

### CD8^+^ T cells release glutamate by a TCR-stimulus-dependent mechanism

In a first set of experiments, we isolated naive CD8^+^ T cells from splenocytes of WT C57BL/6 mice, stimulated them polyclonally with and without anti-CD3/CD28-coated beads at a bead-to-cell ratio of 1:1 in medium containing 2 mM glutamine and determined supernatant glutamate concentrations at distinct time points. Supernatant glutamate concentrations increased exponentially with time and reached levels of about 0.5 mM after 72 hours of bead-stimulation (n = 3 mice, experiments performed in triplicates; Figure 1A). In contrast, in the absence of bead-stimulation, supernatant glutamate concentrations after 72 hours were significantly lower than in its presence (relative supernatant glutamate: bead-stimulation for 72 hours: 1.0 ± 0.01, no bead-stimulation for 72 hours: 0.17 ± 0.04, *P* = 0.002, n = 3 mice, experiments performed in triplicates; Figure 1C). Moreover, supernatant glutamate concentrations determined after 72 hours of stimulation clearly depended on the bead-to-cell ratio as a measure of stimulation intensity (Figure 1B).

**Figure 2:**
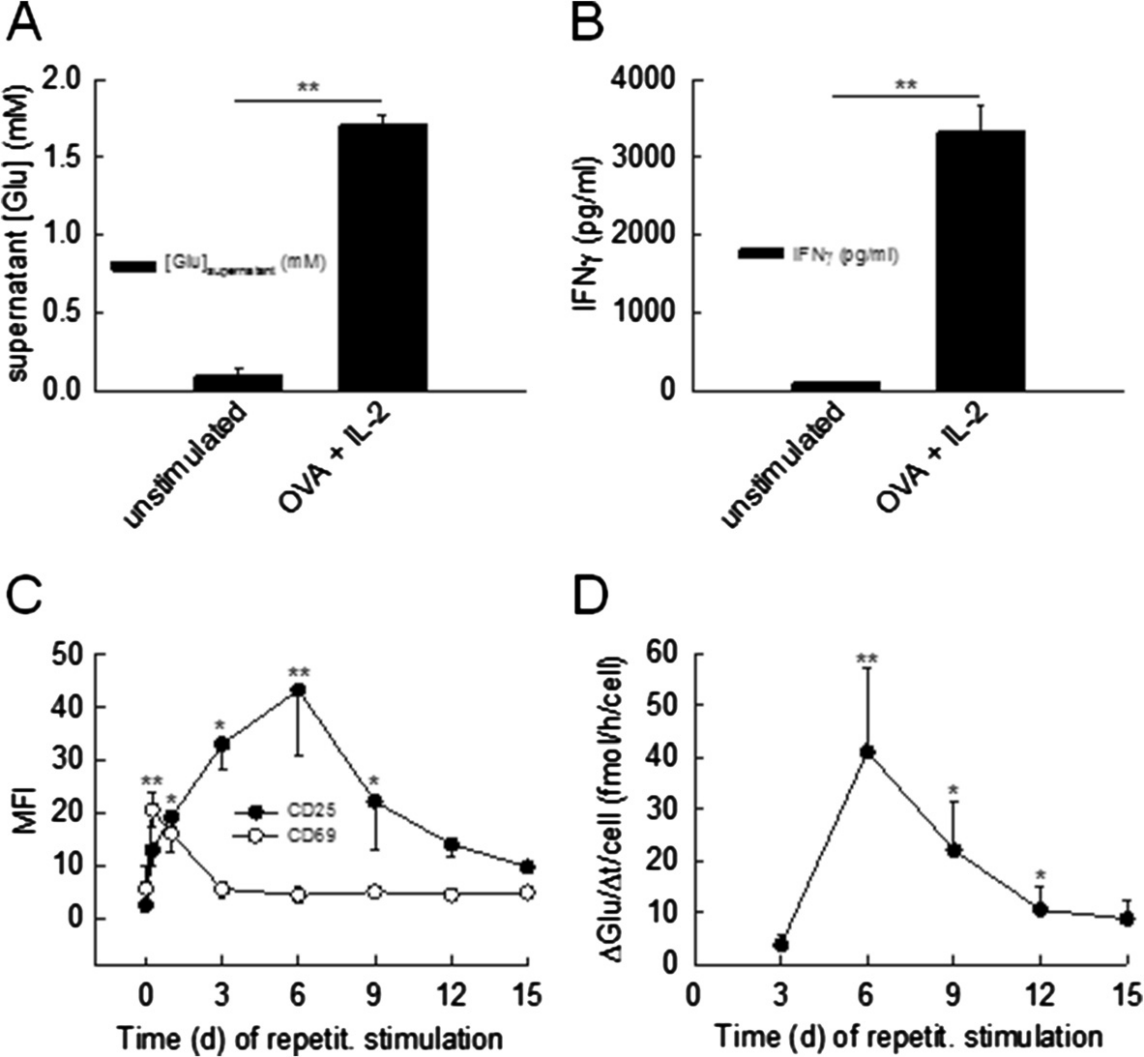
**Upon antigen-dependent stimulation CD8**^**+ **^**T effector cells exhibit higher estimated single cell glutamate release rates than CD8**^**+ **^**T memory cells. (A**, **B)** Supernatant glutamate **(A)** and IFNγ **(B)** concentrations determined after 72 hours of stimulation of splenocytes from OT-I mice bearing a transgenic ovalbumin (OVA)-reactive TCR with or without OVA-peptide_257-264_ (SIINFEKL; 1 nM) and IL-2 (500 IU/ml) (n = 3 mice, experiments performed in triplicates). **(C**, **D)** Time course of T cell activation marker expression (mean fluorescence intensity (MFI) for CD69 and CD25; **(C)** and estimated single cell glutamate secretion rates (∆Glu/∆t/cell; **(D)** during repetitive stimulation of splenocytes from OT-I mice by incubation for time intervals of 72 hours with OVA-peptide_257-264_ (SIINFEKL; 1 nM) and IL-2 (500 IU/ml) (n = 3 mice, experiments performed in triplicates).

As glutamate has important roles in the intermediary metabolism, it is present at millimolar concentrations in the cytosol of most cell types [17]. Cytosolic glutamate may stem from transamination of α-ketoglutarate by glutamate dehydrogenase or from deamination of glutamine by glutaminase [17]. Consistent with predominant synthesis of intracellular glutamate from deamination of glutamine, supernatant glutamate levels were significantly reduced following stimulation of CD8^+^ T cells for 72 hours in glutamine-free medium (relative supernatant glutamate: bead-stimulation for 72 hours in glutamine-containing medium at a bead-to-cell ratio of 1:1: 1.0 ± 0.01, bead-stimulation for 72 hours in glutamine-free medium at a bead-to-cell ratio of 1:1: 0.20 ± 0.05, *P* = 0.003, n = 3 mice, experiments performed in triplicates; Figure 1C).

To address the mechanism of glutamate release, we stimulated CD8^+^ T cells with anti-CD3/CD28-coated beads at a bead-to-cell ratio of 1:1 in the presence and absence of a variety of blockers of non-vesicular glutamate liberation (Figure 1C): L-aminoadipic acid (L-AAA, 2.5 mM) blocking the system X_c_-glutamate-cystine exchanger (Takeuchi *et al*., 2006), 4-(2-butyl-6,7-dichloro-2-cyclopentylindan-1-on-5-yl)oxybutyric acid (DCPIB, 5 μM) blocking volume-sensitive anion channels [18], L-β-threo-benzyl-aspartate (TBOA, 500 μM) blocking excitatory amino acid transporters (EAATs; [19]), carbenoxolone (carben, 10 μM) blocking connexin hemi-channels [13] and pyridoxal phosphate-6-azo(benzene-2,4-disulfonic acid) (PPADS, 100 μM) blocking P_2_× receptors [20]. After 72 hours of stimulation in the presence of each blocker, cell viability was controlled via flow cytometry for annexin V and propidium iodide (PI) uptake. As a consequence of cytotoxic effects, the concentration of carbenoxolone was reduced from 100 μM [13] to 10 μM and that of DCPIB was reduced from 20 μM [18] to 5 μM. Under these experimental conditions the fraction of viable annexin V^-^ PI^-^ cells was always > 90% (Additional file 1: Figure S1D). The presence of blockers did not affect the activation status of CD8^+^ T cells after 72 hours of stimulation as under all conditions the fraction of activated CD8^+^ CD25^+^ T cells was > 95% (Additional file 1: Figure S1C).

None of the tested blockers of non-vesicular glutamate release mechanisms significantly reduced supernatant glutamate levels after 72 hours of CD8^+^ T cell bead-stimulation (relative supernatant glutamate: bead-stimulation for 72 hours: no blocker: 1.0 ± 0.01; TBOA: 0.92 ± 0.05, *P* = 0.129; PPADS: 1.03 ± 0.05, *P* = 0.325; DCPIB: 0.91 ± 0.04, *P* = 0.296; carbenoxolone: 0.96 ± 0.02, *P* = 0.312; n = 3 mice respectively, experiments performed in triplicates; Figure 1C). Moreover, L-AAA caused a significant increase of supernatant glutamate levels (relative supernatant glutamate: bead-stimulation for 72 hours: no blocker: 1.0 ± 0.01; L-AAA: 1.42 ± 0.06, *P* = 0.01; n = 3 mice respectively, experiments performed in triplicates; Figure 1C), consistent with a predominant role of the system Xc-glutamate-cystine exchanger over EAATs for (re-)uptake of extracellular glutamate during bead-stimulation of CD8^+^ T cells.

As we were unable to unambiguously identify a non-vesicular mechanism of glutamate release from stimulated CD8^+^ T cells, we studied the expression regulation of genes essential for vesicular glutamate release during anti-CD3/CD28-bead stimulation of WT CD8^+^ T cells (Figure 1D). Quantitative real-time qRT-PCR analysis revealed an up-regulation of expression levels of glutaminase (3.3 ± 0.9 fold) responsible for the cytosolic conversion of glutamate from glutamine [17]. Moreover, vesicular proton ATPase (3.6 ± 0.1 fold), required for vesicle acidification and vesicular glutamate transporters (VGluT 1: 77.0 ± 12.0 fold; VGluT2: 12.6 ± 7.2 fold; VGluT3: 5.2 ± 1.2 fold; n = 3 mice, all experiments performed in triplicates) required for uptake of cytosolic glutamate into vesicles [21] showed significant expression regulation (Figure 1D). These findings point towards vesicular release as the predominant mechanism of glutamate liberation by polyclonally activated CD 8^+^ T cells.

### Antigen-dependent glutamate release from CD8^+^ T cells varies with repetitive stimulation

To assess whether CD8^+^ T cells also secrete glutamate upon antigen-dependent stimulation, we incubated splenocytes from OT-I mice [5] bearing a transgenic OVA-reactive TCR with OVA-peptide_257-264_ (SIINFEKL; 1 nM) and IL-2 (500 IU/ml). Supernatant glutamate concentrations after 72 hours of incubation with the antigen-peptide were significantly higher than in its absence (supernatant glutamate: antigen-peptide stimulation for 72 hours: 1.70 ± 0.07 mM; no antigen-peptide stimulation for 72 hours: 0.09 ± 0.05 mM, *P* = 0.002, n = 3 mice, experiments performed in triplicates; Figure 2A). The same was true for supernatant IFNγ levels (supernatant IFNγ: antigen-peptide stimulation for 72 hours: 3,325 ± 34 pg/ml; no antigen-peptide stimulation for 72 hours: 92 ± 17 pg/ml, *P* = 0.005, n = 3 mice, experiments performed in triplicates; Figure 2B).

**Figure 3:**
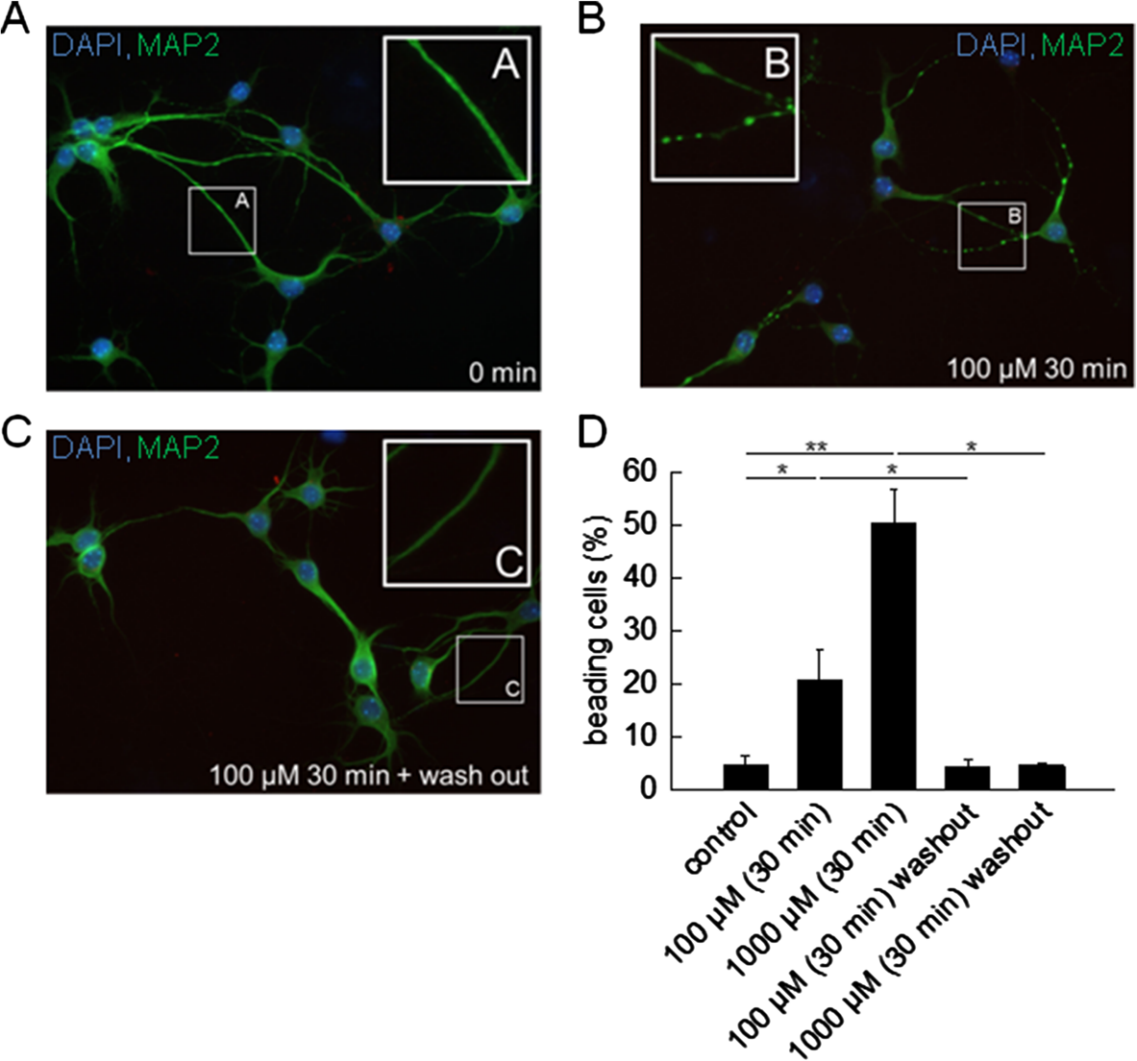
**Neurons exposed to glutamate reversibly and dose-dependently exhibit focal swellings of their neurites, that is neuronal beading. (A**-**D)** L-glutamate exposition (0, 100 and 1,000 μM) for 30 minutes followed by washout for 30 minutes dose-dependently and reversibly increased the proportion of cultured neurons showing focal swellings of their neurites, that is neuronal beading. **(A**-**C)** Representative images showing cultured neurons (MAP2, green; DAPI, blue) before **(A)** and after **(B)** 30 minutes of exposition to 100 μM glutamate as well as after another 30 minutes of wash-out **(C)**. **(D)** Fraction of beading neurons under distinct experimental conditions (n = 5 respectively).

During repetitive antigen-stimulation, CD8^+^ T cells with strong IL-2 receptor α (CD25) expression represent a population of rather short-lived effector cells, whereas those with low CD25 expression form a population of rather long-lived memory cells [22]. To examine an effect of the CD8^+^ T cell activation status on the rate of glutamate release, splenocytes from OT-I mice were repetitively stimulated by incubation for time intervals of 72 hours with OVA-peptide_257-264_ (SIINFEKL; 1 nM) and IL-2 (500 IU/ml) for a total of 15 days. Single CD8^+^ T cell glutamate secretion rates were determined from the supernatant glutamate concentrations and cell numbers at the end of each stimulation interval (see Methods; Figure 2D). In parallel, flow cytometry was repetitively performed for activation markers CD69 and CD25 (Figure 2C). CD69 expression levels peaked after 12 hours of stimulation and went back to baseline levels afterwards (CD69: mean fluorescence intensity (MFI): day 0: 5.6 ± 4.2, day 0.5: 20.6 ± 3.2, *P* = 0.015). In contrast, both CD25 expression levels and single CD8^+^ T cell glutamate secretion rates increased during the two stimulation intervals, peaked at day 6, and subsequently decreased back towards baseline levels (CD25: mean fluorescence intensity (MFI): day 0: 2.5 ± 1.2, day 6: 43.3 ± 12.5, *P* = 0.002; glutamate secretion rate: day 3: 3.8 ± 1.8 fmol/h/cell, day 6: 41.1 ± 16.0 fmol/h/cell, *P* = 0.034; Figure 2C,D) suggesting glutamate liberation as a possible effector mechanism of antigen-activated CD8^+^ T cells.

### Antigen-dependent glutamate release from CD8^+^ T cells causes excitotoxic neuronal cell death during an oligodendrocyte-directed CD8^+^ T cell attack in the CNS gray matter

Glutamate release from activated CD8^+^ T cell might represent an effector mechanism in autoimmune CNS inflammation due to the ubiquitous expression of synaptic and extrasynaptic ionotropic glutamate receptors in neurons [23], which renders them highly sensitive to excitotoxic cell death. Moreover, encephalitogenic CD8^+^ T cells are known to migrate through the CNS parenchyma making short-lasting direct contacts with parenchymal cells in search of their cognate antigen. Upon antigen-recognition, CD8^+^ T cells make long-lasting contacts with their target cells and exert direct and collateral [4,7,12] cytotoxic effects. In the intact CNS parenchyma, glutamate is efficiently cleared from the extracellular space through (re-)uptake by high-affinity glial and neuronal excitatory amino acid transporters (EAATs; [24]). Thus, relevant excitotoxic effects of CD8^+^ T cell-mediated glutamate liberation can only be studied in the intact CNS parenchyma.

**Figure 4:**
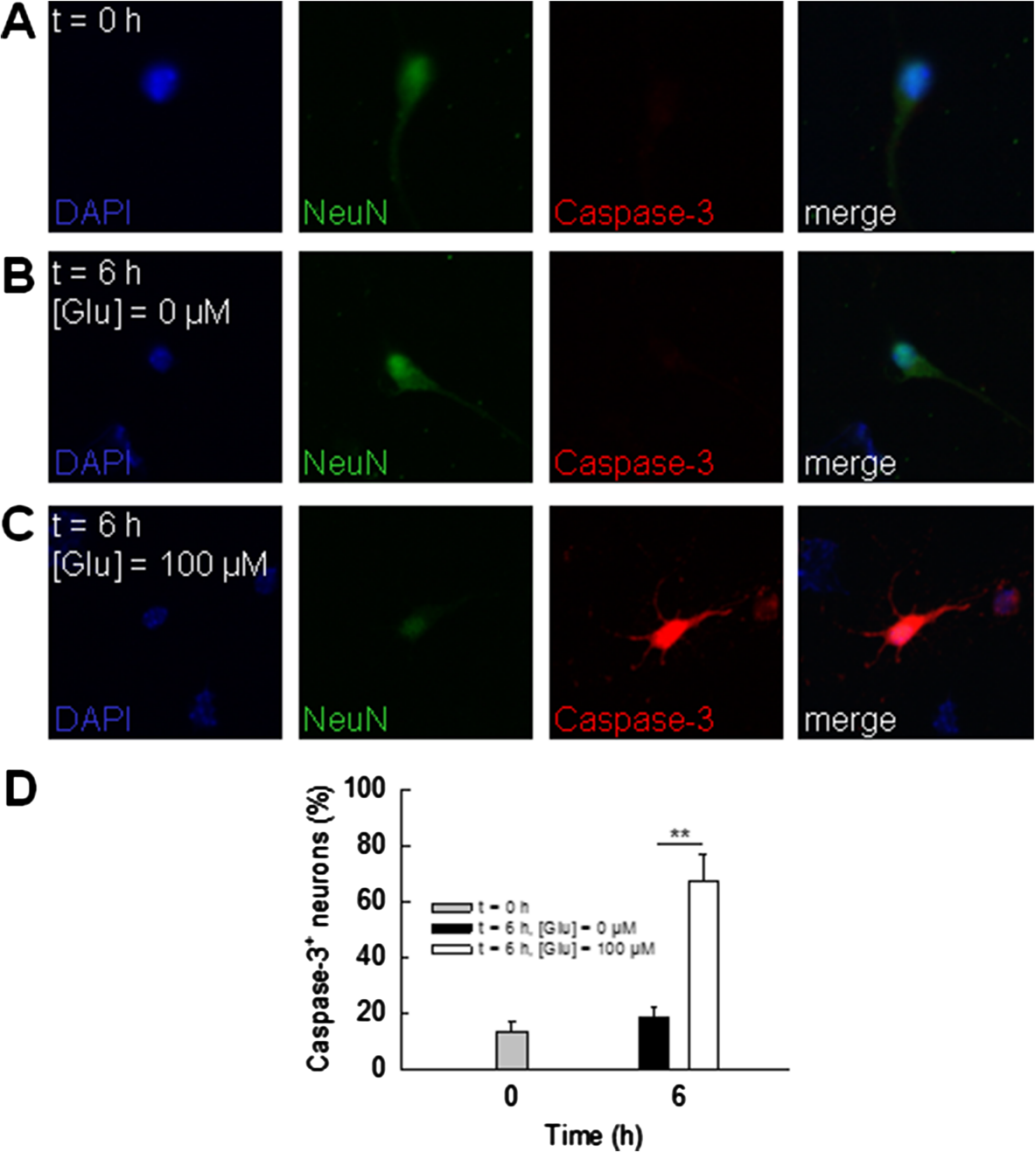
**Caspase-3 activation in neurons exposed to glutamate. (A**, **B)** Representative double staining for activated caspase-3 (red) in cultured neurons (NeuN, green; DAPI, blue) before **(A)** and after exposure to 0 μM **(B)** or 100 μM **(C)** L-glutamate for six hours. **(D)** Fraction of activated caspase-3^+^ neurons under distinct experimental conditions (n = 5 respectively).

As a first step, we tested the glutamate sensitivity of cultured neurons from WT C57BL/6 mice by two independent approaches: (i) cultured neurons were incubated for 30 minutes with standard artificial cerebrospinal fluid (ACSF) supplemented with different concentrations of L-glutamate (0, 100 and 1,000 μM) followed by a washout for 30 minutes with standard ACSF (Figure 3A-C). Glutamate exposition dose-dependently and reversibly increased the proportion of MAP2^+^ neurons showing focal swellings of their neurites, that is neuronal beading [13] (fraction of beading cells: control with 0 μM glutamate: 4.5 ± 2.0%; 100 μM glutamate: 20.7 ± 5.8%, *P* = 0.04, washout: 4.5 ± 1.4%, *P* = 0.04; 1,000 μM glutamate: 50.3 ± 6.3, *P* = 0.003, washout: 4.6 ± 0.6%, *P* = 0.02; n = 5 respectively); (ii) cultured neurons were incubated for six hours with standard ACSF supplemented with or without L-glutamate (100 μM; Figure 4A-D). The proportion of NeuN^+^ neurons staining positive for activated caspase-3 was calculated before and after six hours of incubation as a percentage of all NeuN^+^ cells. After six hours of exposition to glutamate-containing ACSF, the fraction of activated caspase-3^+^ NeuN^+^ neurons was significantly increased compared to incubation with ACSF without glutamate (incubation for 0 hours: 13.2 ± 3.4%; incubation for six hours: ACSF without glutamate: 18.6 ± 3.8%, ACSF with 100 μM glutamate: 67.3 ± 9.6%, *P* = 0.009). Hence, caspase-3 activation occurs as a consequence of exposure of neurons to toxic amounts of glutamate [25-28].

Excitotoxic neuronal cell death is a consequence of excessive stimulation of ionotropic glutamate receptors and can be effectively inhibited by MK801 (blocking NMDA-receptors at a concentration of 10 μM; [29] and NBQX (blocking AMPA-/kainate-receptors at a concentration of 30 μM; [29]).

**Figure 5:**
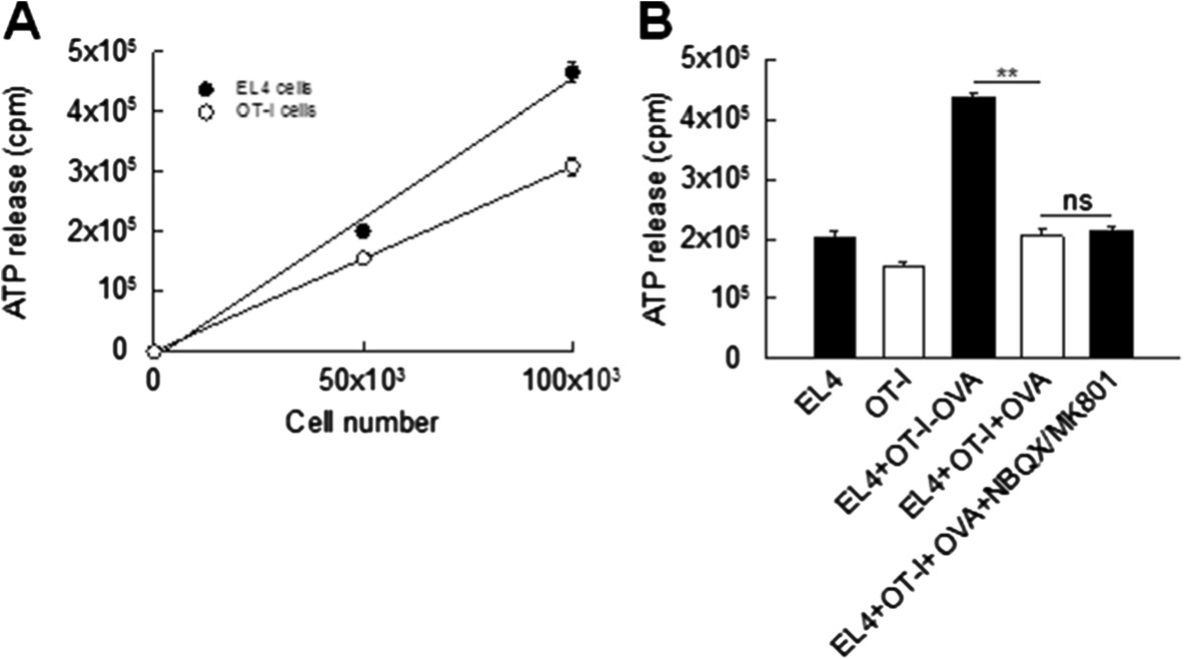
**Ionotropic glutamate receptor blockers NBQX and MK801 do not impact antigen-dependent CD8**^**+ **^**T cell mediated killing of murine lymphoma cells. (A**, **B)** Impact of ionotropic glutamate receptor blockers MK801 (10 μM) and NBQX (30 μM) on the killing behavior of activated OT-I T cells using murine EL-4 lymphoma cells loaded with ovalbumin (OVA)- peptide_257-264_ (SIINFEKL; 1 nM) as target cells (effector-to-target cell ratio 1:1). The amount of ATP in the supernatant following cell lysis was assessed as a parameter of target cell viability. **(A)** Supernatant ATP levels determined from 0, 50,000 and 100,000 EL-4 cells and activated OT-I T cells cultured separately for six hours demonstrate a linear dependence on the number of cultured cells (n = 3 mice, experiments performed in triplicates). **(B)** In the absence of OVA-peptide_257-264_ loading of EL-4 cells (EL4), supernatant ATP levels upon co-culture with activated OT-I T cells (OT-I) were approximately the sum of those obtained by separate culture of both cell types (EL-4 + OT-I-OVA). OVA-peptide_257-264_ loading of EL-4 cells significantly reduced supernatant ATP levels upon co-culture with activated OT-I T cells (EL-4 + OT-I + OVA) for six hours. MK801 and NBQX had no impact on the killing efficacy (EL-4 + OT-I + OVA + MK801/NBQX; n = 3 mice, experiments performed in triplicates for all experimental groups).

First, we tested the impact of MK801 and NBQX at the respective concentrations on the killing behavior of activated OT-I T cells using glutamate-insensitive murine EL-4 lymphoma cells [30] loaded with OVA-peptide_257-264_ (SIINFEKL; 1 nM) as target cells (effector-to-target cell ratio 1:1; [10]; Figure 5). The amount of ATP in the supernatant following cell lysis was assessed as a parameter of target cell viability. As a control, supernatant ATP levels were determined from 0, 50 000 and 100 000 EL-4 cells and activated OT-I T cells cultured separately for six hours (n = 3 mice, experiments performed in triplicates). Under all conditions, supernatant ATP levels showed a linear dependence on the number of cultured cells (Figure 5A). In the absence of OVA-peptide_257-264_ loading of EL-4 cells, supernatant ATP levels upon co-culture with activated OT-I T cells were approximately the sum of those obtained by separate culture of both cell types (EL-4 cells: 200 949 ± 12 298 cpm, OT-I T cells: 154 765 ± 6 131 cpm, EL-4 cells + OT-I T cells - OVA-peptide: 436 011 ± 8 309 cpm) making relevant antigen-independent cytotoxicity unlikely. In contrast, OVA-peptide_257-264_ loading of EL-4 cells significantly reduced supernatant ATP levels upon co-culture with activated OT-I T cells (EL-4 cells + OT-I T cells + OVA-peptide: 204 716 ± 10 553 cpm, *P* < 0.001) consistent with a strong killing efficacy. MK801 and NBQX at the respective concentrations had no impact on the OT-I T cell killing efficacy (EL-4 cells + OT-I T cells + OVA-peptide + MK801/NBQX: 214 982 ± 6 812 cpm, *P* = 0.148, n = 3 mice, experiments performed in triplicates for all experimental groups; Figure 5B).

To exclude relevant self-killing of OT-I T cells due to OVA-peptide_257-264_ loading of their MHC I molecules, we used EG-7 cells as targets, which constitutively present OVA-peptide_257-264_ in the context of MHC I [10] and obtained the same results (EG-7 cells + OT-I T cells: 259 042 ± 24 119 cpm; EG-7 cells + OT-I T cells + MK801/NBQX: 199 698 ± 25 930 cpm, *P* = 0.143, n = 3 mice, experiments performed in triplicates for all experimental groups, data not shown). Hence, glutamate receptor blockers did not impact the killing efficacy as a measure of migration, target cell recognition and exertion of effector functions of activated CD8^+^ T cells. They can thus be used to study the impact of CD8^+^ T cell-mediated glutamate liberation on excitotoxic neuronal cell death within the intact CNS parenchyma.

**Figure 6:**
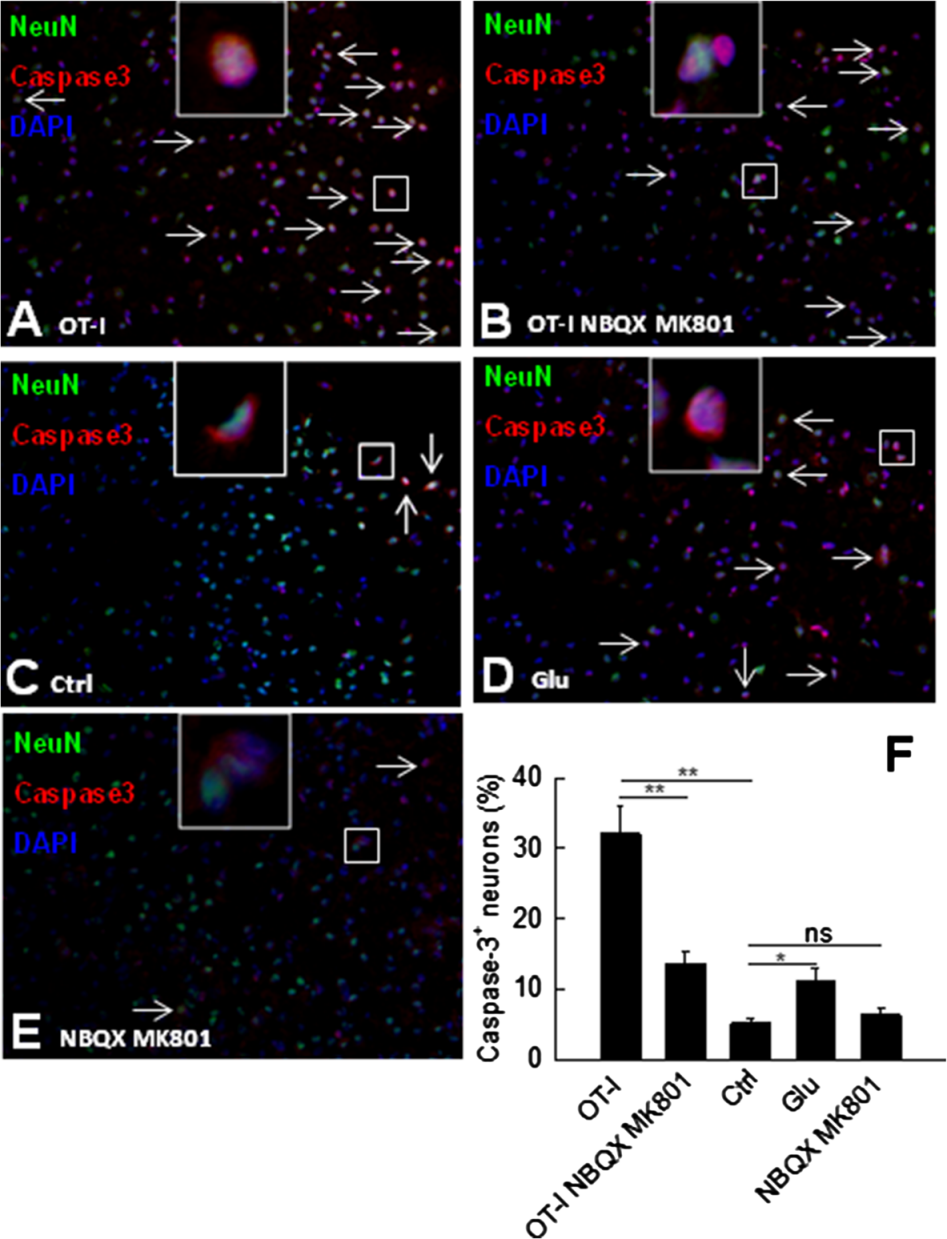
**Antigen-dependent glutamate release from CD8**^**+ **^**T cells causes collateral excitotoxic neuronal cell death during an oligodendrocyte-directed CD8**^**+ **^**T cell attack in the CNS gray matter. (A**-**F)** Activated OT-I T cells were incubated for six hours [5] in acute brain slices from ODC-OVA mice in the absence **(A**, OT-I**)** or presence **(B**, OT-I NBQX MK801**)** of MK801 (10 μM) and NBQX (30 μM), and collateral neuronal cell death (white arrows) in the cortical gray matter was tracked via staining for NeuN (green), DAPI (blue) and activated caspase-3 (red). As controls, acute brain slices from ODC-OVA mice were left untreated **(C**, control**)**, or incubated either with L-glutamate **(D**, Glu, 300 μM**)** or with MK801 and NBQX **(E**, NBQX MK801, 10 μM and 30 μM**)** for six hours. **(F)** Fractions of activated caspase-3^+^ NeuN^+^ neurons detected in the cortical gray matter of acute brain slices from ODC-OVA mice under the distinct experimental conditions (n = 3 mice, each providing three brain slices for all experimental groups).

To this end, we transferred activated OT-I T cells [5] into acute brain slices from mice selectively expressing OVA in oligodendrocytes (ODC-OVA mice [6]) and tracked collateral neuronal cell death in the cortical gray matter via double-staining for NeuN and activated caspase-3 [7,10]. After six hours exposure to activated OT-I T cells, the fraction of activated caspase-3^+^ neurons was significantly increased compared to control conditions without OT-I T cell incubation (OT-I T cells: 32.1 ± 4.0%; control: 5.2 ± 0.8%, *P* = 0.008). Moreover, glutamate receptor blockade with NBQX and MK801 significantly reduced neuronal cell death (OT-I T cells: 32.1 ± 4.0%; OT-I T cells + MK801/NBQX: 13.6 ± 1.9%, *P* < 0.001). Importantly, incubation with NBQX and MK801 did not reduce background levels of neuronal cell death in the absence of incubation with OT-I T cells (MK801/NBQX: 6.4 ± 0.9%, *P* = 0.236) ruling out unspecific inhibition of excitotoxicity in the absence of T cells. Furthermore, incubation for six hours with L-glutamate at a concentration of 100 μM significantly increased the fraction of activated caspase-3^+^ neurons compared to control conditions (glutamate: 11.2 ± 1.8%, *P* = 0.041, n = 3 mice, each providing three brain slices for all experimental groups; Figure 6) corroborating caspase-3 activation in neurons undergoing excitotoxic cell death within the intact CNS parenchyma.

## Discussion

CD8^+^ T cell-mediated neuronal cell death is a relevant pathogenic mechanism in a variety of inflammatory CNS disorders [4]. CD8^+^ T cell cytotoxicity within the CNS is predominantly mediated via two independent pathways depending on the strength of the antigen-signal (that is, the number of antigen-peptide loaded major histocompatibility complex class I (pMHC I) molecules and the affinity of the CD8^+^ T cell receptor (TCR) complex to the pMHC I complex) delivered by the glial or neuronal target cell [31,32]. Strong antigen-signals promote killing via CD8^+^ T cell-mediated perforin and granzyme exocytosis and perforin-dependent delivery of granzymes into the target cell, whereas weak antigen-signals favor killing via interaction of CD8^+^ T cell-derived Fas-ligand (FasL) with the Fas-receptor (Fas) on the target cell surface [31-33]. Moreover, CD8^+^ T cells are able to contribute to inflammatory tissue damage by release of proinflammatory cytokines such as IFNγ and tumor necrosis factor-(TNF)α [34]. These CD8^+^ T cell effector mechanisms impair electrical signaling [12] and cause cell death of neurons as direct and 'collateral’ target cells [4,7].

Glutamate excitotoxicity is known to contribute to neuronal damage in autoimmune CNS inflammation [35-37]. Under inflammatory conditions, glutamate is known to be released from microglia and astrocytes [13,20].

We provide evidence of glutamate release as another crucial effector pathway of CD8^+^ T cells leading to 'collateral’ excitotoxic neuronal degeneration during an oligodendrocyte-directed CD8^+^ T cell attack in the CNS gray matter. Following TCR stimulation, CD8^+^ T cells acquire the molecular repertoire for vesicular glutamate release: (i) they upregulate expression of glutaminase required to generate glutamate via deamination of glutamine and (ii) they upregulate expression of vesicular proton-ATPase and vesicular glutamate transporters required for filling of vesicles with glutamate. In contrast, there was no evidence for non-vesicular mechanisms of glutamate liberation. Furthermore, CD25^high^ CD8^+^ T effector cells exhibit higher estimated single cell glutamate release rates than CD25^low^ CD8^+^ T memory cells underpinning glutamate liberation as a possible effector mechanism of antigen-activated CD8^+^ T cells entering the CNS. Moreover, activated oligodendrocyte-reactive CD25^high^ CD8^+^ T effector cells cause 'collateral’ neuronal cell death within intact cortical gray matter of acute brain slices which can partially be blocked by a combination of ionotropic glutamate receptor antagonists. Hence, glutamate liberation by oligodendrocyte-reactive CD8^+^ T cells is capable of eliciting excitotoxic cell death of neurons despite glutamate reuptake by glia and neurons in the intact CNS gray matter [24]. Encephalitogenic CD8^+^ T cells (in contrast to CD4^+^ T cells) make close contacts with their target cells in the CNS [12] and release proinflammatory cytokines which may reinforce local glutamate excitotoxicity: TNFα is known to reduce the expression of EAATs and detoxifying enzymes in glia cells limiting their glutamate uptake capacity [38,39]. IFNγ has been shown to exert neurotoxicity by gating a calcium-permeable complex of the IFNγ receptor and AMPA glutamate receptor in neurons [40]. Moreover, neuron-reactive CD8^+^ T cells which directly contact neurites induce cytoskeleton breaks with adjacent neuritic spheroids [41] closely resembling neuronal beading upon exposition to glutamate [13]. Importantly, it has been demonstrated that cell death in mature but not immature cultured neurons treated with cytotoxic secretory granules isolated from CD8^+^ T cells can be inhibited by blockers of ionotropic (NMDA- but not AMPA-) but not metabotropic glutamate receptors [30]. As in our case, this effect was neuron-specific as glutamate receptor blocker failed to protect glutamate-insensitive lymphoma cells from secretory granule-induced cytotoxicity [30]. Moreover, blocking of neuronal glutamate reuptake did not significantly enhance killing of neurons elicited by secretory granules, and relevant glutamate concentrations could not be detected in the culture supernatant. This refutes the possibility that significant neuronal (auto-)toxicity was induced by vesicular or non-vesicular release of glutamate from neurons following their perforin-mediated depolarization [12] upon treatment with secretory granules [30]. However, these findings are consistent with storage of glutamate in secretory granules which together with perforin and granzymes elicits neuronal calcium overload and cell death.

## Conclusions

Facing excess numbers of target cells, CNS-invading CD8^+^ T cells may kill neurons either via confined release of cytotoxic effector molecules towards the neuron or via spillover of cytotoxic effector molecules from 'leaky’ immunological synapses and non-confined release by CD8^+^ T cells themselves during serial and simultaneous killing of oligodendrocytes or astrocytes. In both scenarios glutamate-mediated excitotoxicity may contribute to neuronal cell death.

## Abbreviations

ACK: Ammonium-chloride-potassium; ACSF: Artificial cerebrospinal fluid; AMPA: L-alpha-amino-3-hydroxy-5-methyl-4-isoxazole propionate; APC: Allophycocyanin; ATP: Adenosine triphosphate; BSA: Bovine serum albumin; carben: carbenoxolone; CD: Cluster of differentiation; cDNA: complementary DNA; CNS: Central nervous system; DCPIB: 4-(2-butyl-6,7-dichloro-2-cyclopentylindan-1-on-5-yl)oxybutyric acid; DMEM: Dulbecco’s modified Eagle’s medium; EAAT: Excitatory amino acid transporter; Fas: Fas-receptor; FasL: Fas-ligand; FCS: Fetal calf serum; FITC: Fluorescein isothiocyanate; HBSS: Hank’s balanced salt solution; HEPES: 4-(2-hydroxyethyl)-1-piperazineethanesulfonic acid; IFN: Interferon; L-AAA: L-aminoadipic acid; MBP: myelin basic protein; MEM: modified Earl’s medium; MK801: (+)-5-methyl-10,11-dihydro-5H-dibenzo[a,d]cyclohepten-5,10-imine maleate; NBQX: 2,3-dihydroxy-6-nitro-7-sulphamoyl-benzo(F)quinoxaline; NMDA: N-methyl-D-aspartate; ODC: Oligodendrocyte; OT-I: Ovalbumin-reactive CD8^+^ T cells; OVA: Ovalbumin; PBS: Phosphate buffered saline; PE: Phycoerythrin; PFA: Paraformaldehyde; PI: Propidium iodide; PIPES: Piperazine-N,N’-bis(2-ethanesulfonic acid); PPADS: Pyridoxal phosphate-6-azo(benzene-2,4-disulfonic acid); qRT-PCR: Quantitative real-time PCR; RT: Room temperature; SEM: Standard error of the mean; TBOA: L-β-threo-benzyl-aspartate; TCR: T cell receptor; TNF: Tumor necrosis factor; WT: Wild-type.

## Competing interests

The authors declare that they have no competing interests.

## Authors’ contributions

NM, GH, SB, NB, KG and AMH conducted experiments, NM, HW and SGM designed research, supervised experiments and wrote the manuscript. All authors read and approved the final manuscript.

## Supplementary Material

Additional file 1: Figure S1 (A, B) WT CD8^+^ T cells were isolated from splenocytes (see Methods) yielding a purity of about 90% of CD8^+^ cells (A) of which about 90% were CD3^+^ T cells (B) as revealed by standard flow cytometry analysis. (C) The presence of a variety of glutamate release blockers (see text) did not affect the activation status of CD8^+^ T cells after 72 hours (d 3) of CD3/28 bead-stimulation as under all conditions the fraction of activated CD8^+^ CD25^+^ T cells was > 95%, whereas it was < 1% before CD3/28 bead-stimulation (d 0). (D) The presence of a variety of glutamate release blockers (see text) did not impact CD8^+^ T cell viability after 72 hours (d 3) of CD3/28 bead-stimulation as the fraction of viable annexin V^-^ PI^-^ cells was always > 90%. Click here for file
