# Supplementary figures and images for: Excitotoxic neuronal cell death during an oligodendrocyte-directed CD8+ T cell attack in the CNS gray matter
Source: J Neuroinflammation. 2013 Oct 5;10:121. doi: 10.1186/1742-2094-10-121 (PMC3853237; doi:10.1186/1742-2094-10-121)

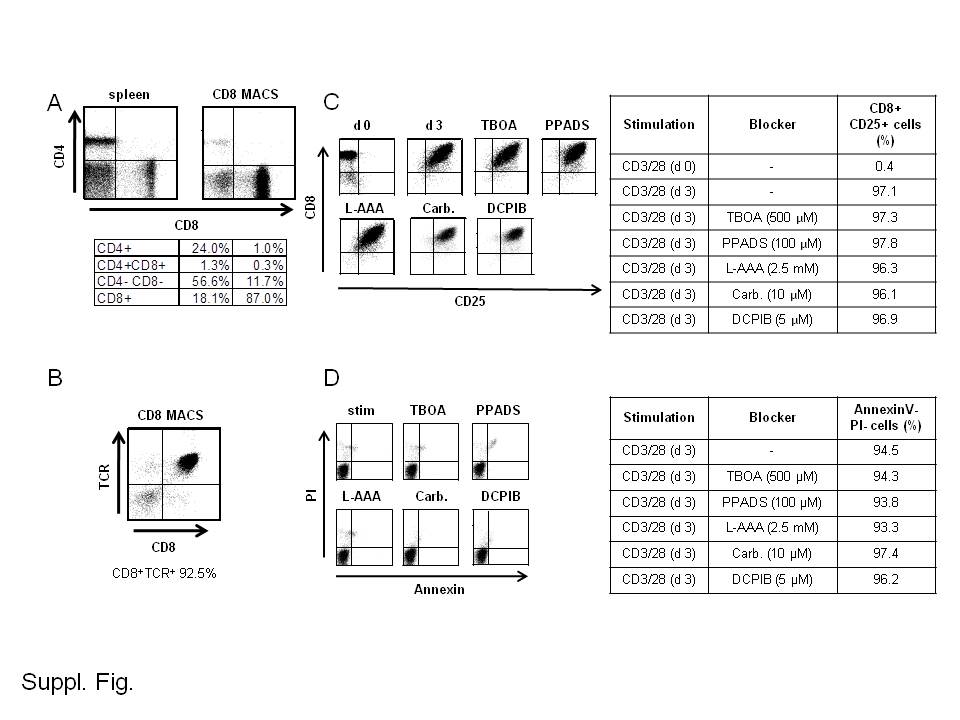

Supplement: Additional file 1: Figure S1 — (A, B) WT CD8+ T cells were isolated from splenocytes (see Methods) yielding a purity of about 90% of CD8+ cells (A) of which about 90% were CD3+ T cells (B) as revealed by standard flow cytometry analysis. (C) The presence of a variety of glutamate release blockers (see text) did not affect the activation status of CD8+ T cells after 72 hours (d 3) of CD3/28 bead-stimulation as under all conditions the fraction of activated CD8+ CD25+ T cells was > 95%, whereas it was < 1% before CD3/28 bead-stimulation (d 0). (D) The presence of a variety of glutamate release blockers (see text) did not impact CD8+ T cell viability after 72 hours (d 3) of CD3/28 bead-stimulation as the fraction of viable annexin V- PI- cells was always > 90%. [file 1742-2094-10-121-S1.tiff]
